# On The (Un)importance of Working Memory in Speech-in-Noise Processing for Listeners with Normal Hearing Thresholds

**DOI:** 10.3389/fpsyg.2016.01268

**Published:** 2016-08-30

**Authors:** Christian Füllgrabe, Stuart Rosen

**Affiliations:** ^1^Medical Research Council Institute of Hearing Research, The University of NottinghamNottingham, UK; ^2^Speech,Hearing and Phonetic Sciences, University College LondonLondon, UK

**Keywords:** working memory, speech perception in noise, aging, normal hearing, hearing loss, supra-threshold auditory processing, sentence identification, reading-span test

## Abstract

With the advent of cognitive hearing science, increased attention has been given to individual differences in cognitive functioning and their explanatory power in accounting for inter-listener variability in the processing of speech in noise (SiN). The psychological construct that has received much interest in recent years is working memory. Empirical evidence indeed confirms the association between WM capacity (WMC) and SiN identification in older hearing-impaired listeners. However, some theoretical models propose that variations in WMC are an important predictor for variations in speech processing abilities in adverse perceptual conditions for *all* listeners, and this notion has become widely accepted within the field. To assess whether WMC also plays a role when listeners without hearing loss process speech in adverse listening conditions, we surveyed published and unpublished studies in which the Reading-Span test (a widely used measure of WMC) was administered in conjunction with a measure of SiN identification, using sentence material routinely used in audiological and hearing research. A meta-analysis revealed that, for young listeners with audiometrically normal hearing, individual variations in WMC are estimated to account for, on average, less than 2% of the variance in SiN identification scores. This result cautions against the (intuitively appealing) assumption that individual variations in WMC are predictive of SiN identification independently of the age and hearing status of the listener.

## Introduction

**FIGURE 1:**
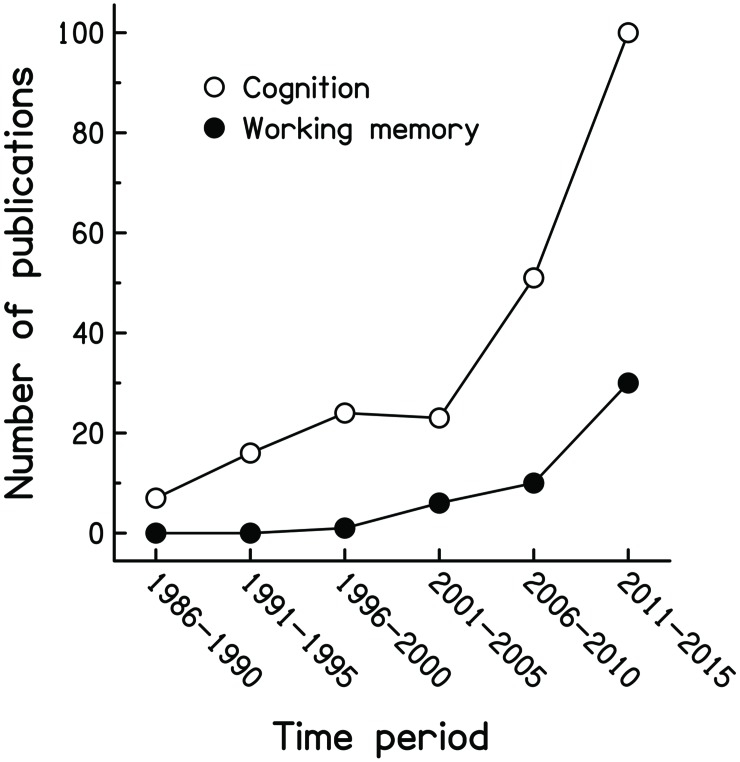
**Publications investigating cognitive abilities and speech processing**. The data points indicate the number of research articles containing in their title or abstract the search terms *speech perception, speech identification, speech intelligibility* or *speech understanding*, and *cognitive, cognition, memory, attention, inhibition* or *speed of processing*, published between 1986 and 2015 in the following journals: Ear and Hearing, International Journal of Audiology (or before 2002: Audiology, British Journal of Audiology and Scandinavian Audiology), Journal of the Acoustical Society of America, Journal of the American Academy of Audiology, Journal of Speech, Language and Hearing Research (or before 1997: Journal of Speech and Hearing Research), Hearing Research and Journal of the Association for Research in Otolaryngology. The filled symbols denote publications featuring *working memory* as the second research term.

Over the past decades, there has been growing interest in the role of individual differences in cognitive functioning in speech processing, reflected by a noticeable increase in the number of scientific publications on this topic (see **Figure 1**). Such work reflects the emergence of the new interdisciplinary research field of Cognitive Hearing Science (e.g., Arlinger et al., 2009), focussing on understanding the interplay of auditory and cognitive processes in speech perception, primarily in adverse circumstances. Not only are key scientific issues at stake, there are also important clinical implications in trying to provide effective rehabilitation to people suffering from problems with spoken communication.

### Working Memory and Its Role in Complex Cognition

Amongst the different cognitive abilities investigated, working memory (WM) has received considerable attention in recent years (see filled symbols in **Figure 1**). WM is considered by many psychologists as a “cognitive primitive,” due to its moderate-to-very-strong associations with different aspects of hot (i.e., emotion-laden; Klein and Boals, 2001) and cold cognition, such as reasoning (Barrouillet, 1996), attentional control (Das-Smaal et al., 1993), comprehension (Daneman and Merikle, 1996), and fact recall and pronoun referencing (Daneman and Carpenter, 1980). Over the years, different definitions have been given for this theoretical construct but it is generally agreed that the capacity of the WM system (WMC) can be reliably assessed by so-called complex span tasks. These require participants to perform a complex activity while concurrently trying to retain new information. For example, in one of the most widely used WM tasks, the Reading-Span (RSpan) test (Baddeley et al., 1985), visually presented sentences have to be read and their plausibility judged, while trying to remember parts of their content for recall after a variable number of sentences.

### The Role of Working Memory in Speech Perception

Given the strong and systematic link between WM and higher-order complex behavior, it is hardly surprising that performance on complex span tasks has also been used to explain individual variability in understanding speech in noise (SiN).

For example, a series of audiological research studies investigated whether individual differences in WMC, measured by the version of the RSpan test developed by Rönnberg et al. (1989), can help predict unaided (Lunner, 2003; Rudner et al., 2011) and aided (Lunner, 2003; Foo et al., 2007; Rudner et al., 2008, 2009, 2011) speech perception in hearing-impaired (HI) listeners, and explain the user-dependent success of different types of signal-processing performed by the hearing aid (e.g., dynamic range or frequency compression; Souza et al., 2015). Mainly moderate, sometimes even strong correlations between SiN identification and RSpan scores were consistently reported. Surprisingly, when referring to these findings to corroborate the role of WM in SiN perception, it is generally not mentioned that the cited studies were conducted with HI listeners who, on average, were aged over 65 years.

Furthermore, on the basis of an extensive review of behavioral studies concerned with the effects of cognitive factors on SiN perception in HI *and* normal-hearing (NH) listeners, Akeroyd (2008) concluded, too, that cognitive functioning is associated with SiN identification, and that WMC, especially when measured by the RSpan test, is the best cognitive predictor. However, these conclusions were based solely on the results from HI listeners (namely the relevant citations in the paragraph above), a fact generally not acknowledged when citing this reference.

A similar assumption that the same crucial cognitive processes are at work in all listeners, independently of their age and hearing status, is made in recent models of speech/language processing (e.g., Rönnberg, 2003; Heald and Nusbaum, 2014). For example, according to the latest instantiation of the Ease of Language Understanding (ELU) model (Rönnberg et al., 2013), any mismatch between the perceptual speech input and the phonological representations stored in long-term memory disrupts automatic lexical retrieval, resulting in the use of explicit, effortful processing mechanisms based on WM. The greater the mismatch, the more effortful listening becomes. Both internal distortions (i.e., related to the integrity of the auditory, linguistic and cognitive systems) and external distortions (e.g., background noise) are supposed to contribute to the mismatch. Consequently, it is assumed within this framework that WMC also plays a role when NH listeners have to process spoken language in acoustically adverse conditions. While no experimental evidence supporting this claim has actually been provided, this notion has become widely accepted within the field.

## Study Survey

To assess the claim that individual variability in WMC accounts for differences in SiN identification even in the absence of hearing loss, we surveyed studies administering the RSpan test^1^ and a measure of SiN identification to participants with audiometrically normal hearing sensitivity.

**FIGURE 2:**
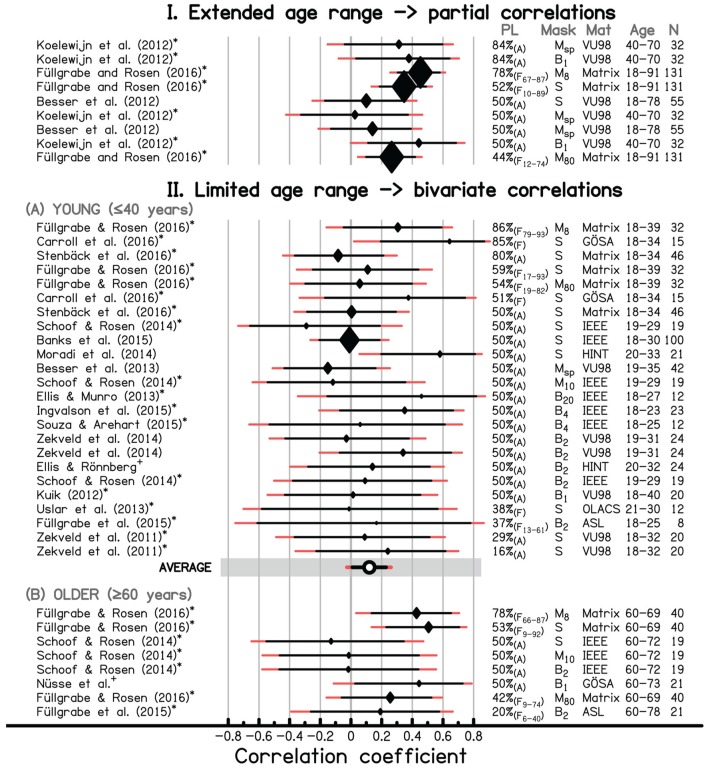
**A forest plot for a meta-analysis of studies investigating the association between WMC and speech-in-“noise” identification in NH listeners after controlling for the effect of age by (I) computing partial correlations or (II) using a limited age range [younger listeners aged ≤40 years (A) *vs.* older listeners aged ≥60 years (B)]**. Shown in the plot are Pearson correlation coefficients (diamonds with their relative sizes indicating the study’s sample size) and associated 95% (black) and 99% (red) confidence intervals. Several studies contributed more than one correlation due to multiple listening conditions, varying in masker type or performance level, also indicated in the Figure (with the exception of the 2014 study by Zekveld et al. (2011) in which the target speech and masker babble were produced by speakers either of the same gender or of different genders). When necessary, the sign of the correlation was changed so that a positive correlation represents better performance on the two tasks. An average for correlations based only on young NH listeners is provided (circle). Also given in the figure are source references (^∗^ indicates re-analyzed published data; + indicates unpublished data, personal communication), experimental conditions (performance level, PL; type of masker, Mask; type of sentence material, Mat) and participant details (age range, Age; number of participants, N). Masker: S – notionally steady noise, M_x_ or M_sp_ – noise modulated by an X-Hz sinusoidal amplitude modulation or a speech envelope, B_x_ – X-talker babble. PL: X%_(A)_ – adaptive procedure tracking the speech reception threshold corresponding to X%-correct identification, X%_(FZ-Y)_ – constant stimuli procedure using several fixed SNRs yielding an overall average performance level of X% with average performance for each of the different SNRs ranging from Z to Y%-correct identification, X%_(F)_ – constant stimuli procedure using a single fixed SNR, yielding an average performance level of X%. In some cases, the modulation depth of the amplitude-modulated noises was only 10%, which is hardly above detection threshold (e.g., Füllgrabe et al., 2005). Therefore, those maskers are labeled as steady rather than modulated.

To ensure consistency with experimental conditions in investigations of HI listeners, only studies presenting sentence material routinely used in audiological and hearing research against spatially co-located background maskers were considered. In addition, we only examined studies in which the effect of age was controlled for, in order to avoid inflated estimates of the correlation between WMC and SiN tasks caused by the tendency for performance in both kinds of tasks to worsen with age. The effect of age was controlled for either by restricting the analysis to a narrow age range, or by statistically partialling out the effect of age when using data from participants across a wider age range. Based on a request posted on the Auditory List^2^ and a general literature search, we were able to compile data from 19 published and unpublished studies that complied with our inclusion criteria^3^. Since several studies measured SiN identification against different types of background maskers or for different performance levels, a total of 41 data sets was entered into the meta-analysis (see **Figure 2**). For each data set, the Pearson correlation coefficient (*r*; diamonds) and associated 95 and 99% confidence intervals (CIs; black and red horizontal lines, respectively) are indicated, as well as the performance level at which the participants were tested, the type of masker, the sentence material^4^, the age range of the sample and the sample size. Within each of the three sections of **Figure 2**, data sets are organized by decreasing performance level (i.e., increasing difficulty). For identical performance levels, data sets are ordered by masker type, representing presumed increasing masker complexity, from “simple” notionally steady noise^5^ through sinusoidally or speech-envelope-modulated noise to speech babble. Interestingly, some of the studies for which the data were reanalyzed on our request (indicated by an asterisk against them) did not even report the correlation between WMC and SiN identification in NH listeners in their original publication.

Across all data sets, the observed *r* values varied widely from -0.29 to 0.64, with almost a quarter of the values being negative, indicating that sometimes low-WMC individuals showed better SiN identification than individuals with high WMC. CIs were rather large, suggesting that studies were underpowered (albeit not necessarily designed to assess this specific relationship), and, in most cases, the intervals included the value zero.

Seemingly in contradiction with the ELU-model prediction of higher WM involvement for speech identification in increasingly adverse listening conditions, there was no obvious trend for more consistent or stronger correlations in more difficult listening conditions (i.e., at lower performance levels). In fact, there is some (descriptive) evidence of stronger associations between WMC and SiN identification in easier listening conditions [see results in section I for the same listeners in high- and low-performance-level conditions in Koelewijn et al. (2012) and Carroll et al. (2016)]. However, this trend was based on results for two performance levels only, and it was not observed consistently across studies (Zekveld et al., 2011; Stenbäck et al., 2016) or even within the same study (Koelewijn et al., 2012).

Moreover, comparisons across different data sets obtained for similar performance levels did not show that inter-individual variability in WMC were more consistently or strongly associated with SiN identification for more complex maskers or target speech, as has previously been speculated (e.g., Rönnberg et al., 2010; Smith and Pichora-Fuller, 2015). For example, for young NH listeners, operating at a performance level of 50%-correct, the correlation for simple relatively predictable HINT sentences presented in a steady noise was 0.58 (Moradi et al., 2014) but only: (i) 0.14 in spectro-temporally and linguistically more complex babble noise (Ellis and Rönnberg, personal communication), and (ii) -0.01 for the linguistically more complex and unpredictable IEEE sentences also presented in steady noise (Banks et al., 2015).

At the same time, the strength of the correlation varied even for studies using very similar test conditions and participant groups. For example, at a performance level of 50%-correct for IEEE sentences presented in a steady noise masker, the correlation for young NH listeners was either -0.29 (Schoof and Rosen, 2014) or -0.01 (Banks et al., 2015). This illustrates the dependence of the results on the particular sample used (and its size) and cautions against basing conclusions as to the role of individual differences in WMC in SiN identification on observations from single small-scale studies.

As there was a sufficiently large number of data sets from studies restricting their sample to young listeners (aged 18–40 years), a random-effects meta-analysis model was used to estimate the average correlation among these studies. This kind of analysis has the advantage not only of assuming that the true treatment effect differs from study to study, but also accounts for the fact that multiple measures can arise from the same study (e.g., where different maskers have been used in the same listeners). The analysis was performed using the R package metafor (Viechtbauer, 2010) and a transformation of the *r* values to Fisher’s *z* scale. Across all 24 data sets, the average *r* value was 0.12. In other words, individual variations in WMC in young people with audiometrically normal hearing are estimated to account for, on average, less than 2% of the variance in SiN identification scores.

Given the considerably smaller number of data sets in each of the two other categories, involving older listeners, we did not compute a summary statistic. However, it is noteworthy that in the largest study included in the survey, using listeners from a wide age range, significant correlations between WMC and SiN identification were found for unmodulated and modulated background noises (see section I of **Figure 2**), and when averaged across maskers, even after partialling out the effects of age and hearing sensitivity (*r* = 0.39; *p* ≤ 0.001; as reported in Füllgrabe and Rosen, 2016). However, separate correlational analyses for each age group in this study revealed that the strength of the association differed across age groups, with the youngest listeners (18–39 years) showing the weakest and a non-significant correlation (*r* = 0.18; *p* = 0.162) while stronger and significant correlations were observed for the middle-aged (40–59 years) to old–old (70–91 years) age groups (all *r* ≥ 0.44; all *p* ≤ 0.011). A linear regression of SiN identification scores against age, RSpan scores and their interaction showed that the slope of the linear dependence of SiN identification performance on RSpan scores indeed increased significantly with age (*p* ≤ 0.001). This illustrates the moderating effect of age on the relationship between WMC and SiN identification, cautioning that the statistical control of the effect of age by computing partial correlations is not necessarily appropriate.

## Discussion and Conclusion

Contrary to common lore and model predictions, this meta-analysis failed to find consistent evidence that, in adverse listening conditions, WMC (as measured by the RSpan test) is a reliable and strong predictor of SiN identification in young listeners with normal hearing thresholds. Recent experimental work on the perception of interrupted speech, another form of signal degradation, is consistent with this finding (Benard et al., 2014; Nagaraj and Knapp, 2015).

It could be argued that the cognitive and speech tests used in the studies surveyed here are suboptimal or inappropriate measures of WMC and SiN processing, respectively (e.g., Besser et al., 2012; Sörqvist and Rönnberg, 2012; Keidser et al., 2015). However, both the conclusions of many empirical studies, showing a link between WMC and SiN processing, and the predictions of the ELU model are based on performance obtained on these very tests.

Another criticism could be made regarding the fact that SiN identification was predominantly assessed for performance levels close to 50% correct, obscuring the possibility that WMC and SiN identification are linked to a greater extent than reported here at other performance levels. Indeed, according to the ELU model, a greater mismatch between sensory and mental representations, and hence a higher involvement of WM-based identification processes, is predicted as speech-to-noise ratios become less favorable. However, this does not seem to be borne out by the collected results. Alternatively, it has also been argued that WM-based restorative processes in older HI (Lunner and Sundewall-Thorén, 2007; Larsby et al., 2008, 2012) and young NH listeners (Stenbäck et al., 2015) might only be effective in conditions where the acoustic signal is not “too” degraded, suggesting a non-monotonic relationship between WMC and SiN identification. While this seems an interesting proposition, the collected results do not indicate the existence of such “sweet spots” for cognitive involvement.

Hence, all things considered, the results of this meta-analysis caution against the (intuitively appealing) assumption that individual variations in WM determine SiN processing in all its forms and independently of the age and hearing status of the listener.

Despite the inconsequential degree to which WMC can predict SiN identification performance in young NH listeners, the reported results should not to be interpreted as evidence against the involvement of cognition in speech and language processing in those listeners *per se*. First, individual differences in WMC have sometimes been shown to explain some of the variability in performance in more linguistically complex tasks, such as the comprehension of conversations (Keidser et al., 2015; but see Smith and Pichora-Fuller, 2015, for contrary results for the comprehension of narratives). Second, different cognitive measures, probing individually the hypothesized sub-processes of WM (e.g., inhibition, shifting, updating; Miyake et al., 2000) or other domain-general cognitive primitives (e.g., processing speed) might prove to be better predictors of SiN processing abilities than the RSpan test (e.g., Sörqvist et al., 2010; Rudner et al., 2011).

It is also important to emphasize that the here reported findings for young NH listeners are not incompatible with the body of evidence showing significant correlations between WMC and SiN identification in primarily older HI listeners. Our own data for NH listeners sampled from across the entire adult lifespan (Füllgrabe and Rosen, 2016) revealed that WMC becomes important for SiN identification from middle age onward, with the oldest listeners (≥70 years) showing the strongest correlation and differing significantly from the youngest age group. One possible explanation for an increasing cognitive involvement in terms of WMC with age, in addition to the loss of audibility, is the accumulation of age-related changes in supra-threshold auditory processing (e.g., sensitivity to temporal-fine-structure and temporal-envelope cues; Schneider and Pichora-Fuller, 2001; Füllgrabe et al., 2003, 2015), sometimes from as early as mid-life (Füllgrabe, 2013). Changes in the coding fidelity of single neurons or across a neural population (Henry and Heinz, 2013; Sergeyenko et al., 2013; Bharadwaj et al., 2014; Lopez-Poveda, 2014), which are not detected by a conventional audiometric assessment, have indeed been associated with degraded sensory representations of the acoustic speech signal. These internal distortions could then call for more WM-based compensatory mechanisms to enable activation of the appropriate representations in long-term memory. Why, however, such age-related internal changes in coding fidelity would result in a greater reliance on WMC for SiN identification than an increase in the amount of energetic and/or informational masking is unclear. Possibly, this discrepancy could be due to secondary changes in the precision of the phonological representations stored in long-term memory, following long-standing auditory processing deficits (e.g., Andersson, 2002; Classon et al., 2013), thus providing a top-down contribution to the mismatch between sensory and mental representations. Clearly, further reflections on the nature and source of listening adversity (see Mattys et al., 2012) are needed to generate oriented hypotheses that can be tested experimentally.

From a clinical perspective, a cognitive assessment (e.g., of WMC) may still prove helpful in improving the prediction of aided SiN identification performance for older audiological patients. Future evidence based on new large samples, independent of those repeatedly investigated in previous studies (Foo et al., 2007; Rudner et al., 2008, 2009, 2011), could further specify the role and importance of cognition in audiological practice.

In conclusion, even though the question of a general *vs.* specialized WM system in language comprehension is not new (Caplan and Waters, 1999) and it has been speculated that differences in tasks and their processing demands activate different sub-components of the WM system, the less-discerning general opinion is that variation in WMC (often assessed by a single measure) can explain differences in performance on a variety of speech tasks. Currently available data from independent research groups do not confirm this assumption for the frequently used task of sentence identification. However, this is not to say that the processing of SiN does not involve a range of cognitive abilities, including WM. For example, it is possible that, even when individual differences exist, the WMC of most individuals is sufficient for the purpose of SiN identification. Systematic efforts are therefore required to establish under which acoustic and linguistic conditions the different cognitive abilities come into play (e.g., Fedorenko, 2014; Smith and Pichora-Fuller, 2015; Heinrich and Knight, 2016). Finally, the results of this meta-analysis clearly highlight the need for a consistent and explicit labeling of the participant characteristics (such as age and hearing status) when reporting results and caution against the untested generalization of research findings from one participant group to another.

## Author Contributions

CF collated, analyzed and plotted the data, and wrote the paper. SR analyzed the data, and revised and commented on the paper.

## Conflict of Interest Statement

The authors declare that the research was conducted in the absence of any commercial or financial relationships that could be construed as a potential conflict of interest. The reviewer MR and handling Editor declared their shared affiliation, and the handling Editor states that the process nevertheless met the standards of a fair and objective review.

## Footnotes

1 Most studies used the RSpan test originally developed by Rönnberg et al. (1989) but some administered a shorter version of the test. However, there seems to be no differences in mean performance between the two test versions (Classon, 2013).

2 http://www.auditory.org/

3 Data from a further two studies were not included in the meta-analysis due to the failure to obtain re-analysed data and the authors’ explicit wish for us not to use their data.

4 Description of the different sentence lists used in the studies entered into the meta-analysis: *ASL* – Adaptive Sentence List (MacLeod and Summerfield, 1990): Predictable simple four- to six-word sentences (e.g., “The boiled egg was soft.”). *HINT* – Hearing In Noise Test (Nilsson et al., 1994; Hällgren et al., 2006): Predictable simple three- to seven-word everyday sentences (e.g., “Strawberry jam is sweet.”). *GÖSA* – Göttinger sentence test (Kollmeier and Wesselkamp, 1997): High-predictability three- to seven-word (mean = 5) everyday sentences (e.g., “The dispute has ended.”). *VU98* – (Versfeld et al., 2000): Eight- or nine-syllable everyday sentences (e.g., “The shop is within walking distance.”). *IEEE* – Institute of Electrical and Electronics Engineers Harvard sentences (Rothauser et al., 1969; Killion et al., 2004): Low-predictability five-keyword sentences (e.g., “A white silk jacket goes with any shoes.”). *OLACS* – Oldenburg Linguistically and Audiologically Controlled Sentences (Uslar et al., 2013): Seven-word sentences of varying linguistic complexity (e.g., “The little boy greets the nice father.” “The farmer, whom the teachers catch, smiles.”). *Matrix* – Matrix sentences (Hagerman, 1982; Vlaming et al., 2011): Low-redundancy five-word sentences with the same syntactic structure (name-verb-number-adjective-object; e.g., “Nina wants some big beds.”). In comparison, the cited investigations involving older HI listeners used HINT and Matrix sentences.

5 Background noise on which no amplitude modulation is impressed is often referred to as a “steady” or “stationary” masker. However, even such notionally steady maskers contain intrinsic random amplitude fluctuations that impede speech perception (Stone et al., 2011, 2012).
